# Comparative evaluation of osseointegration in photo-functionalized versus PRF-coated implants: A split-mouth clinical study

**DOI:** 10.6026/973206300223686

**Published:** 2026-06-30

**Authors:** Devupalli Sudha Madhuri, Papala Sesha Reddy, Reddy Chaithanya, Shaik Mohammed Abbas, T. Naga Sruthi, Sashideepth Reddy

**Affiliations:** 1 Department of Prosthodontics and Crown and Bridge Government Dental College and Hospital, Kadapa, Andhra Pradesh, India

**Keywords:** Photo functionalization, titanium implants, ultraviolet treatment, osseointegration, platelet-rich fibrin (PRF)

## Abstract

Early implant failure and marginal bone loss remain important clinical challenges in implant dentistry and various surface modification techniques have been proposed to enhance osseointegration. Therefore, it is of interest to compare the osseointegration and marginal bone loss of photofunctionalized and PRF-coated implants in completely edentulous mandibular arches. Twelve patients received a total of twenty-four implants, with each patient receiving one UV-activated implant and one PRF-coated implant. Implant stability was evaluated using resonance frequency analysis and marginal bone loss was assessed radiographically over a period of three months. Both groups showed steady improvement in osseointegration, with PRF-coated implants demonstrating slightly higher mean ISQ values, although the difference was not statistically significant. Marginal bone loss was minimal and comparable in both groups, indicating that both techniques performed similarly in terms of early implant stability and bone preservation.

## Background:

The implant-supported overdenture has become a better option than traditional full dentures for fixing completely edentulous mandibular arches. It provides better retention, stability and chewing efficiency, which makes patients happier and improves their quality of life [1]. The long-term success of implant therapy depends a lot on how quickly and stably the implant surface fuses with the surrounding bone, a process called osseointegration. In recent years, a lot of research has gone into changing the surfaces of titanium implants to speed up osseointegration using both physicochemical and biological methods [2, 3, 4]. Titanium dioxide (TiO^2^) is a popular material for implant surface engineering because it is biocompatible and can be activated by light. Photofunctionalization, which uses ultraviolet (UV) light to treat titanium surfaces, has become a popular idea for improving the bioactivity and osteoconductivity of implants [5]. The mechanism of photo functionalization relies on a photocatalytic reaction that creates super hydrophilicity on the TiO^2^ surface, which helps proteins stick to the surface, cells attach to it and cells turn into bone-forming cells [2, 3]. This change lowers the amount of hydrocarbons that build up on the surface during storage and raises the surface energy, which makes it easier for biological fluids and cells to interact with it [4, 6]. Aita *et al.* and Aita *et al.* [5, 6] showed that UV-mediated treatment greatly speeds up the movement, growth and differentiation of human mesenchymal stem cells, which improves the contact between bone and implant *in vivo*. In a similar study, Miyauchi *et al.* [7] found that photofunctionalized nanoscale TiO^2^ layers greatly improved osteoblast adhesion and spreading, which made it easier for bones to attach sooner. Additional research has verified that photofunctionalization modifies both the surface chemistry and the nanoscale topography, enhancing the initial bioactivity of titanium and expediting the biological response at the implant interface [8]. Minamikawa *et al.* [9] noted that photofunctionalized titanium alloys demonstrated enhanced osteoconductivity and bioactivity, underscoring the adaptability of this surface treatment for diverse titanium-based materials. Clinically,Funato *et al.* [10] observed expedited healing times and enhanced implant stability in photofunctionalized implants relative to conventional counterparts, thereby endorsing the integration of this technology into routine practice. Therefore, it is of interest to evaluate the osseointegration in photofunctionalized and PRF-coated implants.

## Methodology:

Twenty-four implants were placed in completely edentulous mandibular arches of patients with sufficient bone height and width. The patients were told about all the treatment options, including the benefits of implant-supported prostheses and they gave their consent before the procedure. The patients were chosen based on certain criteria for inclusion and exclusion that were based on their medical history, clinical evaluation and radiographic assessment. Each patient received two implants: One that was photofunctionalized and one that was PRF-coated. Follow-up exams were done one, two and three months after the implants were put in to check on healing and stability. Two root-form implants were inserted into the mandibular B and D areas of each patient. In Group A, the photofunctionalized implants were activated in a DIO-UV Activator for 20 seconds just before being put in place. In Group B, PRF that had already been made was put into the osteotomy site and the cellular plasma part of PRF was used to wet the implant before it was put into the PRF-filled socket. Using a DIO torque wrench, it was confirmed that all implants had a primary stability of at least 35 N/cm^2^. The study began with a screening phase in which patients were assessed based on the inclusion and exclusion criteria and underwent requisite laboratory tests. A through clinical and radiographic evaluation, encompassing cone-beam computed tomography (CBCT) and intraoral periapical radiographs, was conducted to analyse the morphology of the proposed implant sites and adjacent anatomical structures. Participants received comprehensive information regarding the procedure and alternative treatment options, subsequently providing written consent to confirm voluntary participation. The inclusion criteria consisted of completely edentulous patients possessing sufficient alveolar bone, optimal mucosal health and a commitment to comply with the study protocol. Patients with general contraindications to implant surgery, uncontrolled diabetes, heavy smoking habits, bone disorders, prior head or neck radiation therapy, blood disorders, or temporomandibular joint issues were excluded. A comprehensive medical history was documented for each patient utilising an extensive questionnaire to ascertain any cardiac, renal, gastrointestinal, respiratory, endocrine or neurological disorders, along with bleeding tendencies, allergies, or substance abuse. Dental history was obtained, noting the cause and duration of tooth loss, which included reasons such as caries, periodontal disease, trauma, or neglect. Before putting in the implant, the mouth was checked to make sure that all sources of infection had been treated. The patient's motivation and oral hygiene were also looked at as factors that could affect the long-term outcome. We checked the lymph nodes, glands and soft and hard tissues for problems by doing extraoral and intraoral exams. The patient's vital signs, such as blood pressure, pulse, breathing, temperature, weight and height, were checked and found to be normal. To make sure the patient was fit for surgery; tests were done in the lab, such as a complete blood count, platelet count, haemoglobin level, haematocrit, bleeding time, clotting time, random blood sugar and HIV screening. After these tests, a plan for treatment was made. During the pre-surgical phase, complete dentures were traditionally constructed and subsequently replicated in acrylic to create a surgical template for precise implant placement. A prophylactic dose of 500 mg of amoxicillin was given one hour before the surgery. Patients were rinsed with 0.12% chlorhexidine and the area where the surgery would take place was cleaned with 5% betadine. Under local anesthesia, a full-thickness mucoperiosteal flap was elevated by a mid-crestal incision with vertical buccal releasing incisions. Guided by the surgical template, osteotomy was initiated with a marking drill and completed using progressively larger drills. To achieve optimal primary stability, the final osteotomy was undersized by one drill compared to the planned implant diameter. DIO implants (SLA UFII and UV-activated) were placed with insertion torque values between 30 and 50 Ncm, depending on bone density. Healing abutments were attached and non-absorbable sutures were placed. A post-operative orthopantomogram (OPG) was taken to verify implant positioning and patients were prescribed appropriate medications and postoperative care instructions. Sutures were removed after one week and oral hygiene was reassessed. Follow-up visits were scheduled at one, two, three and six months to evaluate soft and hard tissue changes. After a three-month osseointegration period, healing abutments were removed and prosthetic rehabilitation was performed. Complete dentures were fabricated and the mandibular prosthesis was picked up intraorally using OT Equator Smart Box metal housings and Rhein 83 abutments. The dentures were polished and finalized after occlusal adjustments to ensure even contacts. Patients were advised on maintaining proper oral hygiene and the care of their prosthesis. Implant stability was assessed using resonance frequency analysis (RFA), which operates on the principle that a stronger bone-implant interface results in higher resonance frequencies. The obtained resonance values were expressed as Implant Stability Quotient (ISQ) readings, where higher values indicated better stability. Bone loss around implants was evaluated using radiovisiography (RVG) with the paralleling technique and a RINN-XCP positioning device. A standardized grid was superimposed on the radiographic image and compared with baseline data to determine vertical bone loss and marginal changes around the implants over the follow-up period.

## Results: 

The collected osseointegration data were first entered into Microsoft Excel and later imported into SPSS (Version 26) for statistical analysis. Prior to performing comparative analyses, data normality was assessed using both the Kolmogorov-Smirnov and Shapiro-Wilk tests, which confirmed that the dataset did not follow a normal distribution (p < 0.05) as shown in Table 1. Given this deviation from normality, non-parametric tests were used for subsequent analyses to ensure statistical accuracy and reliability in interpretation.

Both tests demonstrated that p-values were less than 0.05, thereby confirming non-normal distribution and justifying the use of non-parametric tests for analysis. To evaluate temporal changes in osseointegration within each treatment group, the Friedman test (a non-parametric alternative to repeated measures ANOVA) was employed. For intergroup comparisons between UV-coated and PRF-coated implants at corresponding time points, the Wilcoxon signed-rank test was applied due to the paired nature of the split-mouth study design. Descriptive analysis showed that both groups experienced progressive increases in mean osseointegration scores over the study period. The PRF-coated implants consistently exhibited higher mean values at each time interval compared to the UV-coated implants, as summarized in Table 2. As shown in Table 2, both implant groups exhibited progressive improvement in osseointegration over time, with the PRF-coated implants maintaining consistently higher mean values across all time intervals. To further assess these changes, the Friedman test revealed significant differences in osseointegration over time within both groups (p < 0.001), as indicated in Table 3, confirming that osseointegration improved significantly with healing time. In intergroup comparisons using the Wilcoxon signed-rank test, differences between PRF-coated and UV-coated implants were found at certain time intervals (T0, T3, T4) before Bonferroni correction; however, after adjustment (α = 0.01), none of these differences reached statistical significance, as detailed in Table 4. Although PRF-coated implants demonstrated numerically higher osseointegration values at most intervals, these differences were not statistically significant following Bonferroni correction. The mean values of both groups at each time point are shown in Table 5, which further supports that while PRF-treated surfaces consistently yielded higher readings, the variance between groups remained narrow. In addition to osseointegration, peri-implant bone stability was analyzed by assessing marginal bone loss (MBL) at mesial and distal sites three months after prosthetic loading. At the mesial site, both the PRF-coated and UV-activated groups exhibited identical mean bone loss values of 0.383 mm, with a p-value of 1.000, suggesting no statistically significant difference between them. This indicates that both surface modification techniques demonstrated similar performance in minimizing early mesial bone remodeling. At the distal site, the PRF-coated group exhibited a mean bone loss of 0.392 ± 0.138 mm, while the UV-activated group showed a slightly higher mean of 0.425 ± 0.176 mm; however, this difference was not statistically significant (p = 0.611). These findings suggest comparable performance between the two surface treatments in preserving peri-implant bone integrity within the short-term follow-up period, as summarized in Table 6. Overall, the results revealed that both PRF is coating and UV activation techniques effectively promoted osseointegration over time, with PRF-coated implants showing slightly higher mean values. However, the intergroup differences were not statistically significant after correction. Similarly, marginal bone loss analysis indicated that both groups performed comparably in maintaining peri-implant bone stability during the early.

## Discussion:

The current study illustrated that both photofunctionalized and PRF-coated implants exhibited gradual enhancement in osseointegration over time, with PRF-coated implants displaying marginally superior mean stability values, although the difference lacked statistical significance. These results are consistent with earlier research demonstrating that photofunctionalization and autologous platelet concentrates both improve implant-bone integration via unique yet synergistic mechanisms. Suzuki *et al.* [11] and Fugato and Ogawa [12] indicated that photofunctionalized implants exhibited enhanced initial stability and expedited osseointegration, especially in cases of immediate loading, attributed to heightened surface hydrophobicity and bioactivity. Ogawa [13] stressed that UV photo functionalization reverses the biological ageing of titanium by getting rid of surface hydrocarbons, bringing it back to its reactive state and making it easier for cells to attach within minutes of exposure. Simultaneously, platelet-rich fibrin (PRF) has developed as a biologically active autologous substance that can enhance peri-implant healing. Öncü and Alaaddinoglu [14] showed that PRF greatly improves the stability of implants. Boora *et al.* [15] also showed that using PRF during implant placement reduces crestal bone loss and speeds up the healing of soft tissue. The dual mechanism of growth factor release and fibrin matrix formation facilitates angiogenesis and osteogenesis at the bone-implant interface, thereby promoting early osseointegration. Research on UV- modified titanium has demonstrated a consistent improvement in bioactivity and antibacterial properties. De Avila *et al.* [16] demonstrated that UV photo functionalization diminished bacterial adhesion and biofilm formation, thereby fostering a cleaner implant surface conducive to osseointegration. Hirota *et al.* [17] and Kitajima and Ogawa [18] further illustrated that photofunctionalized implants exhibited favourable performance even in adverse bone conditions or cases of low primary stability, indicating a clinical advantage in compromised situations. PRF-based methodologies have progressed, exemplified by Miron *et al.* [19] who presented injectable PRF (i-PRF) as a novel formulation that preserves a high concentration of growth factors, facilitating sustained release and augmented regenerative capacity. A systematic review conducted by Strauss *et al.* [20] validated that the application of PRF markedly enhanced implant stability and diminished marginal bone loss, aligning with the current findings. Additionally, Mehl *et al.* [21] and Hirota *et al.* [22] presented long-term clinical evidence indicating that UV-treated implants demonstrate superior bone-implant contact ratios and sustain stability over several years, further corroborating the efficacy of photo functionalization. Recent research has investigated the synergistic effects of biomimetic coatings and UV activation to enhance the bioactivity of implant surfaces. Dinia *et al.* [23] showed that UV photofunctionalization of biomimetic coatings greatly improved hydrophilicity and the response of osteoblasts, creating a synergistic effect that helped osseointegration. Agrawal and Jaiswal [24] also called i-PRF a biological gem for regenerative dentistry, pointing out how easy, cheap and useful it is in the clinic. Gajiwala *et al.* [25] affirmed that surface modification techniques, including UV activation and PRF application, significantly enhance titanium biocompatibility and osseointegration potential. Kapoor *et al.* [26] compared implants placed with and without PRF and found that the PRF group had much higher implant stability before prosthetic loading. This is very similar to what we found in this study, where PRF-coated implants had higher mean ISQ values. Zhu *et al.* [27] elucidated the advancing array of surface modification techniques, emphasising the significant contributions of both physicochemical (UV activation) and biological (PRF) methods in augmenting osseointegration efficiency. Nakhaei *et al.* [28] elucidated that UV treatment enhances epithelial cell adhesion through decarbonisation, potentially facilitating improved soft-tissue integration around implants. Finally, a recent meta-analysis by Qu *et al.* [29] determined that platelet concentrates markedly improve implant stability and diminish early marginal bone loss, especially during the initial healing phase. The similar results of UV-activated and PRF-coated implants in this study indicate that both techniques are effective. PRF has the extra benefit of being biologically compatible and delivering growth factors, while photo functionalization makes the implant surface very reactive and hydrophilic. These findings collectively emphasise that both surface modifications enhance early osseointegration and safeguard peri-implant bone, rendering them significant clinical adjuncts in modern implant therapy. The present findings demonstrating comparable implant stability and minimal marginal bone loss in both groups are consistent with previous evidence suggesting that biological and surface modification strategies can enhance early implant healing and osseointegration. Carrasco-Labra *et al.* also emphasized the importance of optimizing implant-bone interaction to improve clinical outcomes in implant therapy [30].

## Conclusion

Both photofunctionalization and PRF coating significantly enhanced osseointegration and implant stability over time, with no statistically significant difference between the two treatment modalities. PRF-coated implants demonstrated a trend toward improved stability and marginal bone preservation. Therefore, PRF may serve as a biologically beneficial and cost-effective alternative to UV photofunctionalization for promoting early implant integration.

## Advancement to knowledge:

This study provides clinical evidence comparing osseointegration and marginal bone loss in photo-functionalized and PRF-coated implants using a split-mouth design. The findings contribute to understanding how these surface modification techniques influence implant stability and peri-implant bone preservation, helping clinicians improve implant treatment outcomes.

## Figures and Tables

**Table 1:** Shapiro-wilk and kolmogorov-smirnov tests for normality

**Groups**	**Kolmogorov-Smirnov Sig**	**Shapiro-Wilk Sig**
UV-coated	0.171 (.000)	.932 (.003)
PRF-coated	0.141 (.005)	.930 (.002)

**Table 2:** Mean and Standard Deviation (SD) of Osseointegration Scores

**Group**	**Timepoint**	**Mean ± SD**	**Minimum**	**Maximum**
UV-coated	T0	67.50 ± 5.23	58	74
	T1	64.92 ± 5.53	55	73
	T2	67.17 ± 4.88	58	73
	T3	68.17 ± 5.06	58	73
	T4	69.17 ± 5.20	59	76
PRF-coated	T0	70.58 ± 4.27	63	77
	T1	67.50 ± 4.44	60	72
	T2	69.25 ± 3.77	62	73
	T3	70.92 ± 4.14	61	75
	T4	72.25 ± 3.75	63	76

**Table 3:** Friedman test results for osseointegration across time intervals

**Group**	N	**Chi-square (^2^)**	**DF**	**P-value**	**Interpretation**
UV-coated	12	34.46	4	<0.001	Significant difference over time
PRF-coated	12	34.187	4	< 0.001	Significant difference over time

**Table 4:** Wilcoxon Signed-Rank Test between PRF-Coated (B) and UV-Coated (A) Groups

**Comparison**	**Z-score**	**p-value**
T0 (B0 vs A0)	-2.254	0.024
T1 (B1 vs A1)	-1.261	0.207
T2 (B2 vs A2)	-1.343	0.179
T3 (B3 vs A3)	-2.01	0.044
T4 (B4 vs A4)	-2.425	0.015

**Table 5:** Pairwise comparison of mean osseointegration scores

**Timepoint**	**Group A (UV-coated) Mean ± SD**	**Group B (PRF-coated)Mean ± SD**
T0	67.50 ± 5.23	70.58 ± 4.27
T1	64.92 ± 5.53	67.50 ± 4.44
T2	67.17 ± 4.88	69.25 ± 3.77
T3	68.17 ± 5.06	70.92 ± 4.14
T4	69.17 ± 5.20	72.25 ± 3.75

**Table 6:** Intergroup comparisons of marginal bone loss three months after loading

**Comparison**	**Group**	**N**	**Mean ± SD**	**p-value**
Mesial	PRF-coated	12	0.383 ± 0.111	1
	UV-activated	12	0.383 ± 0.164	
Distal	PRF-coated	12	0.392 ± 0.138	0.611
	UV-activated	12	0.425 ± 0.176	
